# Does fludrocortisone treatment cause hypomagnesemia in children with primary adrenal insufficiency?

**DOI:** 10.3906/sag-2008-167

**Published:** 2021-02-26

**Authors:** İbrahim Mert ERBAŞ, Selda Ayça ALTINCIK, Gönül ÇATLI, Tolga ÜNÜVAR, Bayram ÖZHAN, Ayhan ABACI, Ahmet ANIK

**Affiliations:** 1 Department of Pediatric Endocrinology, Faculty of Medicine, Dokuz Eylül University, İzmir Turkey; 2 Department of Pediatric Endocrinology, Faculty of Medicine, Pamukkale University, Denizli Turkey; 3 Department of Pediatric Endocrinology, Faculty of Medicine, İzmir Kâtip Çelebi University, İzmir Turkey; 4 Department of Pediatric Endocrinology, Faculty of Medicine, Aydın Adnan Menderes University, Aydın Turkey

**Keywords:** Mineralocorticoids, magnesium, magnesuria, pediatrics, primary adrenal insufficiency

## Abstract

**Background/aim:**

Aldosterone is a mineralocorticoid that secreted from adrenal glands and a known factor to increase magnesium excretion by direct and indirect effects on renal tubular cells. Although the frequency of hypomagnesemia was found to be approximately 5% in adult studies, there is no study in the literature investigating the frequency of hypomagnesemia in children by using fludrocortisone, which has a mineralocorticoid activity.

**Materials and methods:**

A multi-center retrospective study was conducted, including children who were under fludrocortisone treatment for primary adrenal insufficiency and applied to participant pediatric endocrinology outpatient clinics.

**Results:**

Forty-three patients (58.1% male, 41.9% prepubertal) included in the study, whose median age was 9.18 (0.61-19) years, and the most common diagnosis among the patients was a salt-wasting form of congenital adrenal hyperplasia (67.4%). Mean serum magnesium level was 2.05 (±0.13) mg/dL, and hypomagnesemia was not observed in any of the patients treated with fludrocortisone. None of the patients had increased urinary excretion of magnesium.

**Conclusion:**

Unlike the studies performed in adults, we could not find any evidence of magnesium wasting effect of fludrocortisone treatment with normal or even high doses in children and adolescents.

## 1. Introduction

Magnesium is an inorganic ion, which is mostly stored at intracellular space, especially in bones. There is not any specific hormone that regulates serum levels of magnesium, homeostasis of which is primarily sustained by gastrointestinal absorption and renal excretion [1]. About 80% of plasma magnesium is filtered through the glomerulus in kidneys and partially reabsorbed at the proximal tubule first. Most of the filtered magnesium is reabsorbed at the thick ascending limb of the loop of Henle, and approximately 5%-10% is reabsorbed at the distal convoluted tubule, depending on the voltage gradient, which is created by potassium secretion into the distal tubule. This process in distal tubule is an essential physiological factor for magnesium balance [2,3].

Aldosterone is a mineralocorticoid that secreted from adrenal glands and has a regulator effect on blood pressure, water, and electrolyte homeostasis. It increases sodium (Na+) reabsorption in both distal, connecting tubules or collecting ducts of the nephrons [4]. Also, aldosterone is known to increase magnesium excretion by direct and indirect effects on renal tubular cells [5]. The transient receptor potential melastatin-7 (TRPM7) is one of the main ion channels thought to be responsible for this effect [6-8]. Although the data for explaining this are very limited and contrasting, there were reported cases of hyperaldosteronism, concomitant with magnesium deficiency and increased renal magnesium excretion [5,9,10].

Fludrocortisone has mineralocorticoid activity and is used in the treatment of primary adrenal insufficiency (PAI) in children. Among the side effects of fludrocortisone, hypertension, and electrolyte disorders are frequently seen [11,12]. The most common electrolyte disorder is hypokalemia and has been reported in about 50% of patients. The frequency of hypomagnesemia was found to be approximately 5% in adult studies [13]. However, there is no study in the literature investigating the frequency of hypomagnesemia in children using fludrocortisone.

In this study, we aimed to investigate the frequency of hypomagnesemia in children who received the diagnosis of PAI and treated with fludrocortisone.

## 2. Materials and methods

A multicenter retrospective study was conducted, including patients aged 0-19 years who were under fludrocortisone treatment for PAI and applied to pediatric endocrinology outpatient clinics between August and December 2019. The diagnostic categories were registered as follows: congenital adrenal hyperplasia, autoimmune adrenalitis, X-linked adrenal hypoplasia congenita, adrenalectomy, and others. Patients who had a transient case of adrenal insufficiency, adrenal dysfunction according to hypothalamic, and pituitary disorders or patients who did not receive fludrocortisone treatment were excluded.

Data including date of birth, age at diagnosis, sex, clinical presentation, treatment modalities and durations, age at the onset of treatment were collected from patients’ clinical records. The following data were obtained from these patients on their clinical follow-up at pediatric endocrinology outpatient clinics: weight (kg), height (m), body mass index (kg/m2), blood pressure (mmHg), pubertal stage according to Tanner [14], serum and urine electrolytes, serum cortisol, adrenocorticotropic hormone (ACTH), parathormone, alkaline phosphatase, 25-OH vitamin D and plasma renin activity. All tests were performed early in the morning between 8-9 AM. The reference values for spot urine calcium and magnesium were 0.9-37.9 mg/dL and 2.09-23.21 mg/dL, respectively. The normal range of spot urine magnesium/creatinine ratio was 0.11-0.21. Plasma renin activity level was defined as high when it was greater than the upper limit for ages given as follows: 37 ng/mL/h under 1-year of age, 10 ng/mL/h for 1 to 3-years, 7 ng/mL/h for 3 to 15-years, 9 ng/mL/h above 15-years of age [15].

Standard deviation scores (SDS) for weight, height, and body mass index were calculated with the online calculator for pediatric endocrinologists (Child Metrics) [16] using the reference created for the Turkish population by Neyzi et al. [17]. Blood pressure measurements were interpreted according to percentile values for the appropriate age, height, and sex [18]. Bone age assessment was performed according to Greulich-Pyle radiographic atlas, with the left-hand wrist radiograph of each case [19].

Primary adrenal insufficiency diagnosis was made according to the criterias as follows: (a) clinical symptoms and signs suggestive of PAI, such as hyperpigmentation, distinctive electrolyte imbalance, salt craving; (b) ACTH plasma levels measured at 8 AM were higher than the reference range for two times, concomitant with a cortisol level of < 140 nmol/L (5 µg/dL); (c) a variant in a gene whose mutation known to cause PAI [20]. The fludrocortisone treatment was given to all patients with PAI, as well as patients with simple virilizing form of congenital adrenal hyperplasia who had elevated plasma renin activity levels or showed symptoms of mineralocorticoid deficiency. The dose of fludrocortisone was considered as a high dose in those who received more than 150 µg/m2/day under 2-years of age, and more than 100 µg/m2/day over 2-years of age [21].

This study was approved by the ethical committee of Aydın Adnan Menderes University Faculty of Medicine (Ethics approval number: 2020/80-20) and performed according to the principles of the Declaration of Helsinki. Signed informed written consent form was not obtained due to the retrospective nature of the study. 

### 2.1. Statistical analyses

All statistical analyses were performed using the SPSS application for Windows version 24.0 (IBM Co., Armonk, NY, USA). Clinical data were presented as number (%), mean±standard deviation for normal distribution and median (minimum-maximum value) for data that were not distributed normally. For statistical analysis, comparisons were performed with the ındependent samples test (student’s t-test) or the Mann–Whitney U test as appropriate. The Pearson correlation test was applied for correlations between the parameters. A P-value of <0.05 was considered statistically significant. 

## 3. Results

Forty-three patients (58.1% male, 41.9% prepubertal) included in the study, whose median age was 9.18 (0.61–19) years, bone age was 10.75 (0.4 - 18.5) years and age of diagnosis was 29 days (3 days–17 years). Anthropometric measurements of the patients were given in Table 1. The most common diagnosis among the patients was congenital adrenal hyperplasia salt-wasting form (67.4%). Other diagnoses were found as follows: simple virilizing form of congenital adrenal hyperplasia (18.6%), X-linked adrenal hypoplasia congenita (4.7%), autoimmune adrenalitis (2.3%), adrenalectomy (2.3%) and other causes of PAI (4.7%). There was a consanguinity between the parents in 23.3% of the patients. 

**Table 1:** Anthropometric measurements and treatment characteristics of the patients.

Age (years)	9.18 (0.61–19)
Bone age (years)	10.75 (0.4–18.5)
Weight SDS	0.29 [(-1.9)–(3.1)]
Height SDS	-0.24 [(-2.7)–(1.9)]
Body mass index SDS	0.36 [(-1.4)–(3.8)]
Systolic blood pressure percentile	81.5 (8–99)
Diastolic blood pressure percentile	88.5 (22–99)
Fludrocortisone treatment duration (years)	6.48 (0.58–15.58)
Fludrocortisone dosage (µg/m2/day)	80.5 (28.09–390.73)
Hydrocortisone dosage (mg/m2/day)	13.08 (± 5.86)

The data were presented as mean±standard deviation for normal distribution and median (minimum–maximum value) for that were not distributed normally. SDS: standard deviation score.

Patients had been treated with fludrocortisone for 6.48 (0.58–15.58) years, and the median dose was 0.1 (0.025–0.2) mg/day corresponded to 80.5 (28.09–390.73) µg/m2/day. Fourteen patients were found as receiving a high dose of fludrocortisone. Mean hydrocortisone treatment dosage was 13.08 (± 5.86) mg/m2/day. Median percentile values of systolic and diastolic blood pressure ​​were calculated as 81.5 (8–99) and 88.5 (22–99), respectively (Table 1).

The serum magnesium level was found to be 2.05 (± 0.13) mg/dL, and hypomagnesemia was not observed in any of the patients treated with fludrocortisone. Mean serum sodium, potassium, and chloride values were in normal ranges. Tests about the bone metabolism were found as follows: serum calcium 9.80 (± 0.47) mg/dL, phosphorus 4.60 (± 0.57) mg/dL, alkaline phosphatase 198.2 (± 84.5) IU/L, 25-OH vitamin D 20.5 (± 6.57) ng/Ml, and parathormone 43.9 (± 18.1) pg/mL. Median levels for ACTH, cortisol, and plasma renin activity were 51.6 (5–1250) pg/mL, 7.90 (0.40–60.1) µg/dL, and 3.01 (0.1–13.5) ng/mL/hour, respectively (Table 2). Plasma renin activity was higher than the upper limit for age in seven patients. None of the patients complained of tetany or involuntary contractions.

**Table 2:** Laboratory findings of the patients.

Parameters	Results	Reference ranges
Serum sodium (mEq/L)	139.4 (± 2.53)	136–145
Serum potassium (mEq/L)	4.38 (± 0.36)	3.5–5.1
Serum chloride (mEq/L)	103.2 (± 2.55)	98–107
Serum calcium (mg/dL)	9.80 (± 0.47)	8.8–10.6
Serum magnesium (mg/dL)	2.05 (± 0.13)	1.8–2.6
Serum phosphorus (mg/dL)	4.60 (± 0.57)	3.7–5.4
Serum alkaline phosphatase (IU/L)	198.2 (± 84.5)	74–390
Serum 25-OH vitamin D (ng/mL)	20.5 (± 6.57)	>20
Serum parathormone (pg/mL)	43.9 (± 18.1)	14–72
Serum ACTH (pg/mL)	51.6 (5–1250)	0–46
Serum cortisol (µg/dL)	7.90 (0.40–60.1)	N/A
Plasma renin activity (ng/mL/h)	3.01 (0.1–13.5)	<37 under 1-year of age, <10 for 1 to 3-years, <7 for 3 to 15-years, <9 above 15-years of age
Spot urine calcium (mg/dL)	4.43 (0.03–14.34)	0.9–37.9
Spot urine calcium/creatinine	0.05 (0.01–0.54)	0–0.21
Spot urine magnesium (mg/dL)	6.89 (1.09–19.53)	2.09–23.21
Spot urine magnesium/creatinine	0.09 (0.02–0.39)	0.11–0.21

The data were presented as mean ± standard deviation for normal distribution and median (minimum–maximum value) for that were not distributed normally. ACTH: adrenocorticotropic hormone; N/A: not available.

The median values ​​of spot urine calcium and magnesium excretion were found as 4.43 (0.03–14.34) and 6.89 (1.09–19.53) mg/dL, respectively. Spot urine calcium/creatinine ratio was calculated as 0.05 (0.01–0.54) and magnesium/creatinine as 0.09 (0.02–0.39) (Table 2). None of the patients had increased urinary excretion of magnesium. There was no significant difference found in urinary or serum magnesium levels between the groups according to plasma renin activity levels (normal or high) (P > 0.05). Although spot urine magnesium level was higher in the group that received high dose fludrocortisone, there was no significant difference between the high dose- or normal dose-fludrocortisone given groups (P > 0.05). Also, serum magnesium and plasma renin activity levels were similar in these two groups (P > 0.05) (Table 3). There was no significant correlation between serum and urinary magnesium levels and hydrocortisone or fludrocortisone dosage, fludrocortisone usage time, or plasma renin activity (P > 0.05).

**Table 3:** Comparison of the serum and urine magnesium levels of the patients grouped according to plasma renin activity and fludrocortisone dosage, and plasma renin activity levels of the patients given normal or high dose of fludrocortisone.

	Patients with normal plasma renin activity (n = 34)	Patients with high plasma renin activity (n = 7)	P
Serum magnesium (mg/dL)	2.05 ± 0.15	2.03 ± 0.06	0.45a
Spot urine magnesium (mg/dL)	6.98 (1.09–19.53)	6.89 (2.65–12.31)	0.79b
	Patients given normal dose of fludrocortisone (n = 29)	Patients given high dose of fludrocortisone (n = 14)	
Serum magnesium (mg/dL)	2.03 ± 0.12	2.11 ± 0.14	0.05a
Spot urine magnesium (mg/dL)	6.58 (1.68–17.0)	7.71 (1.09–19.53)	0.27b
Plasma renin activity (ng/mL/h)	2.75 (0.1–9.22) (n = 27)	3.27 (0.17–13.52)	1.00b

The data were presented as mean±standard deviation for normal distribution and median (minimum–maximum value) for that were not distributed normally. aStudent’s t-test, bMann-Whitney U test, P < 0.05.

## 4. Discussion 

Primary adrenal insufficiency is a rare clinical condition characterized by the insufficient production of the steroid hormones from the adrenal cortex, such as cortisol, aldosterone, and adrenal sex steroids, affecting both sexes equally in childhood [22–24]. The main treatment of PAI is replacing glucocorticoids and mineralocorticoids, principally by hydrocortisone and fludrocortisone [21,22]. The recommended dosage for hydrocortisone ranges between 7.5–15 mg/m2/day, according to the underlying cause [25–27]. Fludrocortisone is the only available mineralocorticoid and used as a single dose of 0.05–0.2 mg/day orally, or with a dose of 100–150 µg/m2/day according to the age of the patient. For children receiving fludrocortisone treatment, blood pressure, electrolytes, and plasma renin activity should be monitored. Normotension, normokalemia, and an upper-normal ranged plasma renin activity indicates adequate dosage for fludrocortisone treatment [21,25,28]. Our treatment doses with hydrocortisone and fludrocortisone were 13.08 mg/m2/day and 80.5 µg/m2/day, respectively. Although there were several patients with hypertension, median values of blood pressure percentiles and plasma renin activity were in normal ranges. Also, serum electrolyte levels of our patients were within the normal ranges.

Magnesium is an intracellular cation, and its serum concentration is kept between 1.8–2.5 mg/dL corresponding to 1% of the total body magnesium [29]. It is held at this level, especially by the balance of reabsorption and excretion mechanisms in the kidneys, which take place mainly at the loop of Henle and proximal or distal tubules. The reabsorption in the distal tubule is essential for the physiological process in fine-tuning of magnesium level [2,8]. Transient receptor potential melastatin-6 (TRPM6) is an essential magnesium transporting channel [30]. An autosomal dominant inherited variant of *KCNA1,* which encodes the voltage-gated potassium channel (Kv1.1) colocalized with TRPM6, reported as a rare cause of isolated hypomagnesemia [31]. In addition, TRPM7 is an omnipresently expressed, aldosterone-responsive ion channel, that is magnesium permeable [6–8]. Aldosterone was reported to increase intracellular magnesium levels and TRPM7 signalling, via nongenomic and genomic signalling cascades [32]. In the genomic pathway, aldosterone augments the genomic expression of proteins involved in electrolyte balance, by binding mineralocorticoid receptors [33,34]. Nongenomic pathways include mineralocorticoid receptor, EGF receptor, and G-protein coupled receptor-dependent mechanisms as well as mineralocorticoid receptor-independent actions [35–39]. Valinsky et al. [4] showed that aldosterone promotes the plasma membrane expression of TRPM7, and therefore TRPM7 flow. These processes occurred via genomic signalling cascades related to mineralocorticoid receptors. They also reported that eplerenone, a mineralocorticoid receptor blocker, inhibited the TRPM7 current [4]. On the other hand, Sontia et al *.* [40] showed that aldosterone reduced expression of renal TRPM7 without effecting TRPM6. These experimental findings suggest that mineralocorticoid receptors play an essential role in magnesium metabolism via TRPMs.

Ichihara et al. [41] showed magnesium as a regulator for aldosterone production. Aldosterone was reported to increase the clearance and excretion of renal magnesium and calcium with normal serum levels [5], although there have been contrary findings that acute effects of mineralocorticoids failed to alter magnesium or calcium excretion [42]. Therefore, it was suggested that increased excretion of magnesium or calcium could be a long term action of mineralocorticoids. Horton et al. [5] reported that urine magnesium levels were decreased after spironolactone administration in patients with primary aldosteronism. Resnick and Laragh [43] reported serum magnesium levels in the normal range but higher than the control group in patients with primary aldosteronism. Also, they suggested that parathyroid hypersecretion is a common finding of primary aldosteronism. Delva et al. [9] showed no difference between levels of total plasma or urinary magnesium, and total plasma calcium within primary aldosteronism and control groups. However, they found intracellular ionized magnesium concentration significantly lower in patients with primary aldosteronism than the control group. They suggested that aldosterone could cause these effects on the magnesium homeostasis by alteration of the sodium-magnesium antiporter activity [9]. Furthermore, Matsouka [10] reported that secondary hyperaldosteronism caused by diuretics increased potassium and magnesium excretion in urine, and also aldosterone antagonists like eplerenone or spironolactone reversed this effect. Despite being treated with fludrocortisone, we found that our patients with PAI had serum and urine magnesium levels in the normal range. Also, their serum or urine calcium and serum parathormone levels were in the normal range, too. 

In an experimental study, after the onset of mineralocorticoid treatment in rats, highly elevated levels of magnesium were detected in urine [44]. Among the side effects of fludrocortisone, the frequency of hypomagnesemia was known to be approximately 5% in adult studies [13]. While this was a classical textbook data, we could not find the related article, so the clinical and biochemical characteristics of these adult patients with hypomagnesemia are not known. O’Connor et al. [45] presented a 27-year-old female case with hypomagnesemia who was diagnosed as an autoimmune polyglandular syndrome and had been given 0.3 mg/day fludrocortisone therapy. In addition, elevated levels of serum magnesium and potassium levels were returned to normal after the onset of mineralocorticoid replacement in two infants with the salt-wasting form of congenital adrenal hyperplasia [46]. Unlike the literature data gained from molecular and clinical studies with adults, we could not find any evidence of hypomagnesemia or renal magnesium wasting in children treated with fludrocortisone. Although this result may be related to the limited number of cases in our study, it suggested that the dose of fludrocortisone we used in our patients was not high enough to increase the excretion of magnesium in the urine as shown in hyperaldosteronism, even in patients that received 3–4 folds higher doses. Therefore, it may be associated with varying responses of mineralocorticoid receptors according to age. 

To our knowledge, this is the first study to investigate the magnesium wasting effect of the fludrocortisone treatment in children with PAI. However, there were some limitations in our study. First of all, it was a retrospective study and the sample size was relatively small. Secondly, we were not able to include an age-matched control group to compare the results with healthy children due to ethical issues. Also, we could not be able to collect a 24–h urine specimen to assess magnesium excretion due to the retrospective nature of the study, which is a more accurate method regarding the circadian rhythm of renal magnesium excretion. Therefore, we used spot urine magnesium levels, which is more practical and compatible with the daily clinical practice, but might be less accurate for measuring fractional excretion of magnesium. Hence, further prospective case-control studies with larger sample sizes and also molecular studies will be helpful to reveal this controversial subject and the underlying pathophysiological mechanisms in childhood.

In this study, unlike the studies performed in adults, we could not find any evidence of magnesium wasting effect of fludrocortisone treatment with normal or even high doses in children and adolescents. Therefore, contrary to the recommendations for adults, we do not recommend routine monitoring of serum magnesium levels in these children. Although aldosterone is known to increase the excretion of magnesium by effecting renal tubular cells directly or indirectly, fludrocortisone did not seem to be causing these effects in appropriate dosages for replacement in children and adolescents. 

## Informed consent

This study was approved by the ethical committee of Aydın Adnan Menderes University Faculty of Medicine (Ethics approval number: 2020/80-20) and performed according to the principles of the Declaration of Helsinki. An informed written consent form was not obtained due to the retrospective nature of the study.
